# 

**Published:** 1897-01

**Authors:** 


### LIST OF CONTRIBUTORS TO VOLUME XVIII.

Andrews, R. R., D.D.S.
Arrington, B. F.
Avery, Otis, D.D.S.
Ball, M. V., M.D.
Barrett, W. C., M.D., D.D.S.
Bethel, L. P., D.D.S., M.D.
Brigiotti, J. G.
Brockway, Albert H., M.D.S.
Burchard, Henry H., M.D., D.D.S.
Cassidy, J. S., D.D.S.
Catching, Dr. B. H.
Cheever, David W., M.D.
Cheney, Charles D.
Custer, L. E., B.S., D.D.S.
Davenport, S. E., D.D.S.
Davenport, Wm. Slocum, D.D.S.
Elliott, William St. George, Jr.
Fillebrown, Dr. Thomas.
Flickinger, Adam, D.D.S.
Gethins, James L.
Gilliams, J. S., M.D., D.D.S.
Girdwood, John, D.D.S., L.D.S.
Grant, George F., D.M.D.
Guilford, S. H., D.D.S.
Hildreth, J. L., M.D.
Howe, J. Morgan, M.D., M.D.S.
Jack, Louis, D.D.S.
Jones, W. H., D.D.S.
Leffmann, Henry, M.D., D.D.S.
Maxfield, George A., D.D.S.
Mills, Dr. G. A.
Mills, Dr. S. Alden.
McManus, Charles, D.D.S.
McManus, James, D.D.S.
Palmer, S. B., M.D.S.
Peeso, Frederick A., D.D.S.
Porter, Dr. A. H.
Price, Weston A.
Register, Dr. H. C.
Roberts, Howard E., D.D.S.
Rollins, William.
Shaw, Louis, M.D.
Swarts, Gardner T., M.D.
Talbot, Eugene S., M.D., D.D.S.
Trueman, William H., D.D.S.
Truman, James, D.D.S.
Werner, J. G. W., D.M.D.
Williams, Jacob L., M.D.

